# Developer perspectives on the ethics of AI-driven neural implants: a qualitative study

**DOI:** 10.1038/s41598-024-58535-4

**Published:** 2024-04-03

**Authors:** Odile C. van Stuijvenberg, Marike L. D. Broekman, Samantha E. C. Wolff, Annelien L. Bredenoord, Karin R. Jongsma

**Affiliations:** 1 Department of Bioethics and Health Humanities, Julius Center, University Medical Center Utrecht, Utrecht University, 3508 GA Utrecht, The Netherlands; 2 Department of Neurosurgery, Haaglanden Medical Center, 2512 VA The Hague, The Netherlands; 3 Department of Neurosurgery, Leiden University Medical Center, 2333 ZA Leiden, The Netherlands; 4 Erasmus School of Philosophy, Erasmus University Rotterdam, 3062 PA Rotterdam, The Netherlands; 5 Netherlands Institute for Neuroscience, 1105 BA Amsterdam, The Netherlands

**Keywords:** Locked-in-syndrome, Amyotrophic lateral sclerosis, Blindness, Deafness, Neuromodulation, Ethics, Biotechnology, Neuroscience, Neurology, Engineering

## Abstract

Convergence of neural implants with artificial intelligence (AI) presents opportunities for the development of novel neural implants and improvement of existing neurotechnologies. While such technological innovation carries great promise for the restoration of neurological functions, they also raise ethical challenges. Developers of AI-driven neural implants possess valuable knowledge on the possibilities, limitations and challenges raised by these innovations; yet their perspectives are underrepresented in academic literature. This study aims to explore perspectives of developers of neurotechnology to outline ethical implications of three AI-driven neural implants: a cochlear implant, a visual neural implant, and a motor intention decoding speech-brain-computer-interface. We conducted semi-structured focus groups with developers (n = 19) of AI-driven neural implants. Respondents shared ethically relevant considerations about AI-driven neural implants that we clustered into three themes: (1) design aspects; (2) challenges in clinical trials; (3) impact on users and society. Developers considered accuracy and reliability of AI-driven neural implants conditional for users’ safety, authenticity, and mental privacy. These needs were magnified by the convergence with AI. Yet, the need for accuracy and reliability may also conflict with potential benefits of AI in terms of efficiency and complex data interpretation. We discuss strategies to mitigate these challenges.

## Introduction

Neural implants for human rehabilitation, cognitive restoration and enhancements are expected to be some of the most rapidly evolving innovations within the field of neurotechnology^1,2^. Using brain-stimulating and brain-decoding techniques, neural implants are invasive brain-computer-interfaces (BCIs) developed to restore limited bodily (e.g. motor, sensory) functionality. Recent developments in software technology, including new signal processing techniques and machine learning, are among the most significant contributions to this rapid advancement^1^. As artificial intelligence (AI) systems become more complex, efficient and faster, there is an increase in development of neuroscience applications^3^. In neural implants, AI can be used to improve signal processing techniques, including the interpretation of electrical signals in the brain^1,4–6^. These significant developments in neurotechnology could lead to improved long-term results and better quality of life (QoL) for those with neurological conditions.

Examples of these emerging neural implants are an AI-driven cochlear implant (AI-CI) to restore or establish auditory function (Box 1), an AI-driven visual neural implant (AI-VNI) to restore visual function (Box 2), and an AI-driven implanted speech-brain-computer-interface (AI-speech-BCI) to enable communication in patients suffering from motor neuron disease, including locked-in-syndrome (LIS) (Box 3). Though these three highly advanced neurotechnologies are currently in varying technology readiness levels, clinical applications of these technologies are fast-approaching.

These technological advancements are paired with ethical questions. In the ethical literature, concerns about autonomy, informed consent, participant selection in clinical research, responsibility for actions, privacy and data-security, potential surgical risks and questions regarding costs and accessibility of neurotechnologies have often been described^7,8^. The convergence of AI with neurotechnologies expands existing and introduces new ethics concerns, including concerns regarding agency and identity, mental privacy, augmentation, accuracy, transparency and biases^3,9^.

As developers are at the forefront of these technological advancements, their views on possibilities, limitations and challenges of these technological innovations are an important source for ethical analysis. Moreover, developers’ perceptions may influence both implicit and explicit design choices and therefore play an important role in shaping these technologies. So far, however, little is known about developers’ perspectives on emerging AI-driven neural implants. To our knowledge, only few qualitative studies with developers of neural implants have been conducted in the past, one study focusing specifically on user-centered design in BCI-research^10^, and two studies focused on rehabilitation specialists for BCIs^11,12^. None of these studies have focused on the convergence of neurotechnology with AI.

In this study we therefore aimed to explore perspectives of developers of neurotechnology to outline ethical implications of three AI-driven neural implants: a CI, VNI and a speech-BCI.

Box 1: the AI-driven cochlear implant (AI-CI) AI technology is currently being used to improve the listening experience of CI-users by developing personalized, and context-aware noise suppression and speech enhancement in CI speech processors. These novel machine learning techniques are developed to optimize the signal, perform auditory scene analysis, and combine binaural signals to enhance speech understanding in noise and to follow target voices in complex scenes, which will impact the overall sound and its perceived quality.

Box 2: the AI-driven visual neural implant (AI-VNI) The VNI is a wireless implanted BCI that aims to restore visual function in blind individuals by generating artificial visual perceptions in the user. Footage from a camera worn by the user is processed and analyzed using an AI-software on a processor carried by the user and converted to information for interfacing with the brain. Through weak electrical stimulation of the visual cortex by probes that cause minimal tissue-damage, an artificial percept of light, called a “phosphene”, is induced. Based on the visual input recorded by the camera, multiple phosphenes can be used to build up a visual percept. The AI application of the VNI may have a second function for automatic scene selection, recognizing the context the user is in and selecting the correct settings to convert the camera footage to phosphene vision that contains the most important visual information in that context.

Box 3: the AI-driven speech-BCI (AI-speech-BCI) The speech-BCI aims to restore the ability to communicate in individuals with LIS due to, for example, motor neuron diseases such as amyotrophic lateral sclerosis (ALS). This type of BCI typically involves an implantable multichannel system that aims to record the signal changes associated with attempts to speak from the motor cortex, and to translate these signals to written text on a computer screen and/or a synthetic computer voice for communication. The decoding process will be performed using AI software, and predictive language models may be used to boost performance.

## Methods and analysis

To explore the perspectives of AI-driven neural implant developers, we conducted a focus group (FG) study. FGs are semi-structured discussions in which a specific set of issues is explored in groups of 4–12 people^13^.

### Ethics statement

This study was carried out in accordance with relevant guidelines and regulations. The study was submitted to the Research Ethics Committee of the University Medical Center Utrecht for review before initiation of the research. The committee determined that the study was exempt from the Medical Research Involving Humans Act (research proposal no. 21/477). Written consent was obtained from all participants in the study. Results were reported using the Consolidated criteria for reporting qualitative research (COREQ) checklist^13^.

### Data collection

Data were collected through semi-structured FGs, conducted between March and June 2022. A semi-structured topic list was used to ensure that important topics were discussed in all FGs, while also allowing room for respondents to discuss or emphasize topics that were of perceived relevance. The topic list was based on review of current bioethics literature and expert knowledge of the research team. Topics that were discussed included developers’ expectations and hopes regarding the technology, important design choices to be made in its development, concerns and risks, conditions for clinical translation and the potential impact on impact their field of work. The full topic list can be found in Supplementary Table 1.

A total of four FGs including in total 19 respondents were conducted (Table 1). FGs were led by a member of the research team trained in qualitative research (OCVS, KRJ) and field notes were made by an observer (KRJ, OCVS, AVDB). FGs lasted on average 1 h and 22 min and were conducted via MS-Teams in either Dutch or English. FGs were recorded and transcribed verbatim.

**Table 1:** Respondent demographics.

Demographic	
Male	*n* = 12
Female	*n* = 7
Age (average)	46.1 years
Career	
Professor	*n* = 3
Senior researchers* (e.g. assistant professor, associate professor, postdoc)	*n* = 8
Junior researchers/PhD candidate	*n* = 2
Senior clinician	*n* = 4
Industry management	*n* = 1
Patient participation expert	*n* = 1
Developer of	
AI-driven cochlear implant	*n* = 6
AI-driven visual neural implant	*n* = 8
AI-driven speech-BCI	*n* = 5

*If developers had both research and clinical careers, they were classified under ‘researchers’.

### Recruitment

We recruited respondents that currently are or have been in the past professionally involved in research and/or development of neural implants (i.e., cochlear implants, visual implants including cortical and retinal implants, and speech-BCIs). We approached academic researchers, researchers at neurotechnology companies, clinicians, rehabilitation specialists, psychologists, and other relevant professionals. Participants were recruited via the research team’s professional network of the INTENSE consortium. For FG 1, FG 2 and FG 3 developers were recruited based on recommendations of researchers in this field (i.e., snowball sampling) and their expertise on the topic. For FG 4 a call for participation was shared by email in the network of researchers developing the speech-BCI, and participants were recruited based on their willingness to participate and expertise on the topic.

Because of the wide variety of disciplines relevant for the development of the VNI, we organized two focus groups including developers of VNIs (FG2 and FG3) (Table 2). FG2 included respondents involved in early stages of development (i.e., hardware- and software development and preclinical trials). FG3 included respondents that had been involved in the clinical implementation of a retinal implant, and who were likely to be involved in the future clinical trials of the VNI.

**Table 2:** Focus group format.

Focus group identifier	Technology of topic	Number of participants
FG 1	AI-driven cochlear implant	6
FG 2	AI-driven visual neural implant	4
FG 3	AI-driven visual neural implant	4
FG 4	AI-driven speech-BCI	5
Total		19

In total, 22 participants consented to participation, of which three eventually did not participate because of conflicting schedules. Respondents were approached by OCVS via email and provided with an information letter and informed consent forms before the start of the FG. Participants were asked for their written consent prior to the interview. Recruitment was ended when data saturation was reached (i.e., when additional FGs did not lead to the identification of relevant new themes)^14^.

### Data analysis

The pseudonymized transcripts were analyzed thematically^15^. OCVS developed initial codes based on the topic list, familiarizations with the data and discussion in the research team. OCVS then coded the transcripts using NVivo12 software. KRJ then critically reviewed a sample of the coded transcripts. These were subsequently systematically reviewed for supporting or conflicting evidence concerning emerging themes and codes. Final thematic coding was based on consensus between OCVS and KRJ. Representative quotes were chosen to illustrate themes and translated into English where necessary. Illustrative quotes can be found in Supplementary Table 2, 3 and 4. In our study we reached saturation in terms that subsequent conversations no longer brought up new issues (‘coding saturation’) and the formulated themes were sufficiently understood by the team (‘meaning saturation’)^14^. Saturation was reached prior to coding for guaranteeing inter-coder reliability).

## Results

Respondents addressed a range of considerations important in the development of AI-driven neural implants. These considerations were clustered around three themes: (1) design aspects; (2) challenges in clinical trials; and (3) impact on users and society.

### Design aspects

Respondents discussed aspects of the design of AI-driven neural implant that were considered important to the development of a successful technology, expressing both aims and challenges.

#### Design aims

Respondents defined the aim of developing a technology that is an actual improvement for users compared to the current gold standard (i.e. AI-VNI vs a white cane, AI-CI vs hearing aids, speech BCI vs eye-tracker (Q1a). Respondents argued that improvements beyond the gold standard could also mean that these technologies could help those currently not served by existing technologies (Q1b), are more ‘future proof’ (i.e. compatible with later-generation AI-applications) (Q1c), or more user-friendly.

User-friendliness was discussed by respondents in terms of ‘usability’. For the AI-VNI, usability was for instance referred to in terms of wearability of the device (e.g. a wireless design) and for the AI-CI in terms of automation (i.e. where AI-technology functions in the background without user interference) (Q1d). Respondents involved in the development of the AI-speech-BCI extensively discussed the importance of usability, in terms of having a good user-interface (Q1e), speed, and in terms of versatility, which was phrased as its applicability in a variety of contexts (Q1f), and its ability to allow for more natural communication compared to communication aids that work with a yes/no system (Q1g). Such a high level of usability could also contribute to reduce frustration of users. Developers of the AI-speech-BCI therefore argued the value of co-creation of the technology with users by including their input in the design (Q1h).

A second important design consideration was the reliability and accuracy of the devices. This was considered important because of foreseen and unforeseen circumstances (e.g. in brain activity) under which these devices will need to function. Respondents therefore expressed the wish to design a device that functions both safely and as intended (Q1i).

#### Design challenges

To fulfill these design aims, respondents discussed challenges to overcome, including epistemic (i.e., knowledge-based) uncertainties regarding the development of AI-driven neural implants. Respondents all stated that knowledge gaps exist regarding technological possibilities and biological mechanisms underlying brain-decoding and brain-modulation, which challenges technological progress (Q1j). Specifically, for the AI-VNI open questions remain regarding the materials for a durable product (as concerns exist on the lifetime of the electrodes) (Q1k), the optimal number of electrodes to be implanted, possibilities for making the device wireless (Q1l), and on how psychophysics and stimulation relates to perception (Q1m). Developers of the AI-CI reported the challenge of the technology achieving its intended goal (i.e., noise reduction that improves listening experience for CI-users) in real-life settings, primarily due to the breadth (i.e., variation) and complexity of (auditory) input (Q1n). Auditory complex situations (e.g., a concert) in which users could select which audio-stream to focus on (e.g., music or conversation), require optimization of all these types of input (Q1o). This challenge also persists in speech-input, because of the spectrum and variety of speech between contexts (e.g., a schoolground or an office meeting) as well as between different languages (Q1p). Adding to this complexity, good functioning of the algorithm in the AI-CI (i.e., reducing noise) may not necessarily mean optimal functioning for the user, as complete noise-reduction in a noisy environment may also lead to an undesirable distortion of voices (Q1q). An individually optimized algorithm was suggested as a potential solution but was considered unfeasible with current technologies (Q1r).

### Challenges in clinical trials

Several challenges were mentioned regarding the clinical translation of AI-driven neural implants to the first application in humans and in clinical trials. These include the risks involved in surgical implantation, selection of suitable trial participants based on parameters such as the demandingness of trial participation, and post-trial abandonment of technologies. These topics were only discussed by respondents involved in the development of the AI-VNI and the AI-speech-BCI. It was argued that AI-CI does not impose new health safety risks for the user and will not require new clinical trials, as this technology is compatible with current CIs.

#### Surgical risks

Respondents stated that proving safety of the neural implants would be the primary focus of clinical trials (Q2a). Besides the known risks of invasive brain surgery (Q2b), other risks related to implantation were mentioned. For the AI-VNI for example, the choice of number of electrodes to be implanted was discussed, which needs to be based on the trade-off between the resolution required for functional visual function and the risks of implanting a device of a certain size. A higher resolution could thus mean a higher level of invasiveness (Q2c), introducing a trade-off between benefits and risks. Naturally, surgical risks are also foreseen for the explantation of these devices.

#### Selection of trial participants

Respondents all agreed that a careful selection of suitable trial candidates is warranted for trials with neural implants because of the demandingness of trial participation. Developers of the AI-VNI discussed that a large time-investment is required for trial participation, due to tests, rehabilitation, training, and travel time to the specialized research centers. They therefore argued that the life-phase of a potential participant may be of relevance in screening and recruitment as it is important to know what impact trial participation will have on their everyday lives, and how much potential candidates are willing (and able) to rearrange their lives (Q2d). In addition, developers of the AI-speech-BCI argued that the demandingness of the trial warrants the inclusion of participants who are highly motivated (Q2e). They discussed that this is often the case for ALS patients as these are generally in the midst of their lives at the time of diagnosis, making them motivated to contribute to science, or because they hope to benefit from participation themselves (Q2f). Developers of the AI-speech-BCI also expressed having the intention to continue these studies for many years. They therefore reported to generally prefer trial participants that already have, or at a later stage would, opt for invasive ventilation and reanimation. Not only because of the risks involved in surgery, but also, they argued, as these choices show that participants are motivated to continue their lives (Q2g). Adding to the demandingness of trial participation for AI-driven neural implants, respondents mentioned the potential media-coverage. As this can have a large impact on trial participants and their private lives, it was suggested that media coverage of these trials should be streamlined and coordinated and that participants should be offered psychological support (Q2h).

Respondents further argued that management of expectations is highly important in AI-driven neural implant trials, both on the expected benefits of the treatment and on the ending of the trial. However, views diverged between developers of the AI-VNI and AI-speech-BCI. Developers of the AI-VNI argued that participants should be selected who fully understand that the development of the AI-VNI is still at an (early) stage of research, and who are not too eager to recover or expect to see again (Q2i), to avoid therapeutic misconception. Respondents therefore suggested an extensive screening process, including psychological tests (Q2j). They also stressed the importance of language in the management of expectations. A respondent for instance argued for using the word “perceive”, rather than “see” in communications with (potential) trial participants of the AI-VNI, to lower expectations of full recovery (Q2k). They argued it to be important that participants are reasonable and accepting of their current state of blindness, as the prototype system of the AI-VNI in the first clinical trial is likely to be temporarily implanted (Q2l) and stressed the importance of informing participants about possible explantation of the device at the end of the trial (Q2m). Also, if devices cannot be explanted (e.g. because of surgical risks) this should be communicated clearly to the participants as this may also have long-term (safety) consequences (Q2n).

In contrast, developers of the AI-speech-BCI preferred to provide some direct benefit to participants, though emphasizing that no guarantees could be made for such benefits (Q2o). They described this as a reason for including patients with a progressive disease such as ALS, as they felt that they could still help these patients (Q2p). Yet, they argued that it should be clearly explained that there is always the risk that a trial is terminated because of a lack of effectiveness or too many side-effects, as this is in fact quite common in this pioneering phase of research (Q2q). Still, respondents reported that even in cases without actual benefit of the device, trial participation could have a positive effect on participants as they may feel good about contributing to society in this way (Q2r).

#### Post-trial abandonment of technologies

Respondents also discussed the risk of ‘abandonment of technologies’ in case of (premature) discontinuation of a trial. In such cases developers stop (further) support and development of the technology, even though trial participants may be benefitting from them.

A developer of the AI-speech-BCI argued that such post-trial abandonment of a technology could be harmful for users. They used an example of a tetraplegic participant of a BCI-exoskeleton trial who had to stop using the device at the end of the trial. The respondent described the sense of shock and loss they had observed in the participant, who had experienced benefit from the device after a long and intensive training period (Q2s). Another respondent added that such abandonment of a technology goes against the principles outlined in the Declaration of Helsinki, and that they therefore had built in provisions for post-trial access in their research trial protocols (Q2t). Notably, a respondent working on the AI-VNI questioned the portrayal of a lack of post-trial access provision in first-in-human studies as blameworthy practices of companies due to unacceptable patient harm. Instead, they argued it to be a grey area, because participants are generally told many times that trial participation may not result in personal benefit and still consent to participation (Q2u).

However, other developers of the AI-VNI equally stressed the importance of continuity of care and support for trial participants and users, also in cases of bankruptcy of the developing company. One respondent argued for the responsibility of developers to hedge the risk of abandonment of the technology in case of bankruptcy. They suggested that arrangements should be made similar to those for the automobile industry, which demand that producers ensure that replacement parts remain available for at least 12 years (Q2v). Respondents, however, also acknowledged challenges related to a post-trial period of continued access, including support for older versions of a neurotechnology (Q2w). Several other approaches to deal with post-trial abandonment of neural implants were discussed, including the development of a support network of researchers for remaining users of devices or the requirement for developers to take out insurance to cover costs of explantation (Q2x). However, legal issues may prevent third parties from repairing these medical devices (Q2y), and the desirability and possibility for explantation of the device may differ per user (age) group, depending on the risks involved (Q2z).

### Impact on users and society

Finally, respondents discussed the impact of the AI-driven neural implants on users and society. Impact on users included the ultimate aim of the devices to promote the independence of its users, the reduction of user-control that may be caused by the integration of AI, and potential implications for users’ autonomy, safety and mental privacy. Impact on society included privacy concerns on a societal level, the importance of societal acceptance, reimbursement of these expensive technologies as an important hurdle for their availability, as well as their potential positive economic effects.

#### Impact on users

##### Promoting independence

All respondents argued that a successful technology would be one that enables users to regain some of their independence (Q3a, Q3b). For the AI-VNI, regaining independence was articulated as improvement in orientation and mobility (Q3c), and in communication (e.g. by enabling users to decide when to interact with others) (Q3d). An AI-VNI could allow users to become more socially engaged (Q3e), reduce a sense of isolation (Q3f), increase self-esteem (Q3g), and improve a sense of agency (Q3h). Respondents argued that improved communication in AI-CI users would allow them to more pro-actively take part in life (Q3i) and develop their functioning and perspectives on the job market (e.g. by being able to partake in meetings) (Q3j). Developers of the AI-speech-BCI similarly stated that regaining speech communication can contribute to a user’s ability to be autonomous and independent and can contribute to their quality of life (QoL) (Q3k). They explained that patients with progressive ALS are an important target population for the AI-speech-BCI, as the device will improve their outlook on being able to communicate independently also when their disease progresses, invasive ventilation is needed, and current assistive technology becomes more difficult to use. This prolonged independence could improve their QoL and the desire to remain alive (Q3l).

##### Predictive algorithms and user-control

Though AI-driven neural implants have the potential to improve the user’s independence and autonomy, it was argued that user-control over the devices may be reduced because of the predictive algorithms counteracting this effect. Regarding the AI-CI, for instance, respondents argued that ideally a system would function without interference of the user, requiring the AI-based scene selector to predict what the user wants to hear, and in which context (Q3m). Yet, if any mistakes are made in this prediction, the user is hindered rather than helped (Q3n), making accurate and reliable functioning important. One respondent argued that allowing users to choose and switch to what they want to listen to, as hearing people can also do, would be the preferable option, as they felt this would install a sense of control in users (Q3o).

For the AI-speech-BCI, a respondent similarly argued that mistakes in the predictive function of AI could introduce a risk for the user’s autonomy as the use of speech and language models in the decoding process may lead to the expression of sentences that are not in fact specifically what the user wanted to say, and biases of the AI model may leak into the performance of the device (Q3p). For the AI-VNI, user-control may similarly be reduced as the use of an AI-system for visual data-processing makes it difficult for the user to learn what intelligence the AI lacks and what visual information is missed by the system because of that. This may be easier to notice if more simple technology (e.g. edge detection) would be used to convert the camera imaging to the phosphene vision (Q3q). Developers of the AI-CI argued that preferences regarding user-control likely vary between users, also depending on the level of control they are accustomed to having using current (hearing) aids (Q3r). They believed the technology should be offered in a way that suits all patients (Q3s).

##### User-safety

Accurate and reliable functioning of AI-driven neural implants was also deemed important for user-safety. For users of the AI-VNI, misleading or incomplete visual perceptions whilst navigating a busy road could cause serious safety issues, as would also be the case if the AI-CI would wrongfully suppress the sound of a car approaching. Therefore, a high level of accuracy and reliability is also needed for the device to be trustworthy to the user (Q3t). Additionally, questions about responsibility for actions may arise in case of inaccurate or unreliable functioning of an algorithm in neural implanted devices (Q3u).

##### User-privacy

The use of AI in neural implants also introduces a risk for data-security and for privacy, as discussed by developers of the AI-CI and AI-speech-BCI.

A first concern arises from the option of processing data of neural implants on an external server. Optimal training of an individual AI in the AI-CI would require processing of recorded surrounding sounds, including personal conversations. Respondents argued that this could introduce both ethical and General Data Protection Regulation (GDPR) issues, depending on how exactly the system is implemented (Q3v). Though once the AI-processor is trained and integrated in the device, full audio recordings may no longer need to be shared with an external server, small scene fragments will still need to be shared for AI-driven scene characterization (Q3w).

A second privacy concern was brought forward by developers of the AI-speech-BCI. Though also mentioning similar concerns to AI-CI developers, stating that computation and processing of data on a cloud system or outsourcing data in other ways comes with privacy risks (including a risk of hacking) (Q3x), they also introduced a different privacy issue. A respondent namely pointed out the AI-speech-BCI may potentially be able to read into thoughts or internal monologues of users when attempting to decode (motor) speech intentions (Q3y). This could introduce issues for users’ mental privacy.

#### Societal impact

##### Privacy of non-users

Concerns regarding privacy also exist at a societal level. For example, (auditory) data processing for the use of the AI-CI will concern data not only from the consenting user but also from persons in their surroundings (Q3z), meaning that their privacy may too be harmed. Respondents however argued that similar (auditory) data recording and online data processing already happens in daily consumer products, such as smartphones or smart speakers, which many people choose to use (Q3aa). Where respondents first used this as an argument for the societal acceptability of such practices, they later added that, for instance, Facebook is sued for such issues of data sharing. To avoid “a Zuckerberg effect”, they argued it might be interesting to investigate how to do this in a more ethically sound way for neural implants (Q3bb). One respondent did note that for a medical device such as the AI-CI the law is stricter than for consumer products (Q3cc) and referred to existing legislation that already forbids the collection of data from which a person can be identified without their consent; making that only parameters get transmitted in the codex rather than full audio signals (Q3dd). However, another respondent argued that besides legal interpretations of privacy in these devices, the perception of [a loss of] privacy is also morally relevant (Q3ee).

##### Societal acceptance

Respondents argued that societal acceptance of neural implants was important as it defines a successful technology (Q3ff), and is conditional to generate funding from companies and investors (Q3gg). A successful AI-VNI could also generate hope for people that treatments are available if they become blind later in life (Q3hh). However, a respondent involved in the development of the AI-speech-BCI warned that in reality the development of these technologies is very slow. Raising too much hope could therefore result in societal disappointment, potentially negatively affecting societal and patients’ acceptance (Q3ii).

##### Costs and availability

Concerns related to such hope were the costs and availability of the technology. Once the technology is fully developed, its availability to potential users largely depends on reimbursement decisions. The small size of the trials performed, the effectiveness compared to other assistive technologies, the small target population, and expected high price of the devices challenge a positive outcome of these decisions (Q3jj). Developers of the AI-CI therefore argued that cost-efficacy is essential to the development of a successful technology (Q3kk). However, though initial costs of these devices may be high, developers of the AI-VNI and the AI-CI also argued for the positive economic impact of these devices. They argued that these technologies could contribute to reducing societal costs by reducing long-term healthcare costs for users (e.g., by a reduction in required rehabilitation support or decrease in hospitalization due to improved health and a reduced risk of falling) and by enabling users to take part in society more equally, and access school and work environments (Q3ll, Q3mm).

## Discussion

Various calls for empirical research on ethical aspects of BCIs have been made in the past decade^7,8^. Our study responds to these calls by shedding light on ethically relevant considerations of developers of AI-driven neural implants, which we clustered into three themes: (1) design aspects; (2) challenges in clinical trials; and (3) impact on users and society. In what follows, we will discuss our findings in light of the broader (empirical) literature.

### Aims, uncertainties and hurdles

The convergence of AI with the field of neurotechnology allows for improvements in signal processing techniques^1^, outperforming humans in decoding and encoding of neural signals in brain-decoding devices like speech-BCIs^4^, optimizing speech and signal-to-noise processing in CIs^6^, and optimizing complex image processing to corresponding electrical stimulation patterns in VNIs^16^.

In this study we found that developers of these AI-driven neural implants defined the aims of their development on multiple levels: as developing a technology that accurately and reliably processes data input for brain-stimulation or in brain-decoding, that provides an improvement over existing (lower tech) aids, that improves independence in users, that is accepted by society, and that is available to those who need them. Worth noting is that needs of potential users were sometimes phrased in an ‘ableist’ manner (e.g. these technologies should support potential users to function in society as it is). This same finding was also reported by Sample et al.^11^ in a qualitative study with rehabilitation professionals. Further insights into whether the perceived needs of developers match those of potential users are pressingly needed. User-centered-design has been proposed to reduce this translational gap, yet Sullivan et al.^10^ also report that though BCI researchers in their study welcomed user-input, they also expressed uncertainty on several aspects of user-centered-design including methodological skepticism. More research on how users can successfully engage in BCI development is thus required.

In our study there was broad agreement amongst respondents that technological development is currently hampered by epistemic uncertainties, including questions on relevant underlying biological mechanisms and technologically feasibilities. Other empirical studies analyzing perspectives of professionals working on neural implants reported additional epistemic uncertainties, including the surgical technique of BCI implantation and potential short- and long-term complications^17^, and the proportionality of risks and benefits of the treatment^18,19^.

Another important open question highlighted in our study was how clinical trials for (AI-driven) neural implants should end. Questions about explantation of neural implants at the end of a trial are a topic of debate, as amongst others the proportionality of risks and benefits remain unclear. It is currently uncertain how users may be affected by device removal^8,20–23^, and long-term safety of neural implants is poorly understood^24–28^. Moreover, a lack of consensus existed regarding appropriate arrangements for continued access to neural implants post-trial, which is currently an important topic of scholarly debate^24,29–33^.

Still, when these technologies eventually will reach the market, financial hurdles may exist for their availability to many potential users. Consideration of these financial aspects in the design of the devices, as well as exploration of reimbursement strategies, are conditional to these technologies positively impacting users and society.

### Accuracy & reliability

It was notable that throughout our study respondents repeatedly emphasized technological characteristics of these devices, especially accuracy and reliability. This may come as no surprise, as accuracy (in addition to speed) is often used as a performance measure for the effectiveness of BCIs^34^. Yet, the suitability of accuracy as a performance measure for BCIs is also debated, as other aspects of the technology such as the level of usability were also argued to be able to constitute benefit. This finding echoes the need for a usable design of BCIs as reported by rehabilitation specialists in the study of Nijboer et al.^12^. Nijboer et al.^19^ argued that though effectiveness may be a necessary prerequisite for benefit, it also depends on the user’s individual preference. Moghimi et al.^34^ argue that BCI performance measures should be modified to individuals with disabilities and the context in which the device needs to operate, also including personal factors such as the nature of disability and environmental factors such as physical, social, and attitudinal issues. The importance attributed to accuracy and reliability of neural implants by developers was also magnified by the convergence with AI. In particular because of the perceived safety risks of inaccurate or unreliable functioning of predictive functions in the AI-CI and AI-VNI, and the potential implications for the autonomy of users of the AI-speech-BCI in case of inaccuracies or biases in the predictive functions. Still, these risks and potential implications do need to be weighed with the potential benefits of AI-technology in these devices. For instance, though AI-based decoding strategies in AI-speech-BCIs have a risk of overshadowing user’s intentions and causing semantic inaccuracies that challenge users’ authenticity, they do have the important potential benefit of improving efficiency and speed of communication, which is an important aim of these devices^35,36^. Yet, our study also showed that developers expect challenges relating to accuracy and reliability to be further exacerbated when these neurotechnologies are translated from the controlled clinical environment to the complexity of daily life. Wolpaw et al.^37^ have argued that “the realization of high reliability and accuracy is perhaps the most difficult and critical challenge now facing BCI research and development” (p.15).

### Mental privacy

The convergence of neurotechnologies with AI-technology was also argued to introduce privacy concerns, as discussed by developers of the AI-CI and AI-speech-BCI. This is not unexpected as privacy issues in AI-technologies are an important topic of scholarly debate, often referring to risks in data-protection and data-security^38^. Notably, no privacy issues were mentioned by developers of the AI-VNI, even though the literature on visual neuroprostheses has described these issues^7^. Yet, for the brain-decoding AI-speech-BCI, privacy was not only discussed in terms of data security but also in terms of risks for user’s mental privacy (i.e. if internal monologues are decoded that users do not intend to externalize). Such concerns on mental privacy of neurotechnology users have increasingly gained attention in the neuroethics community. It has for instance been argued that current legislative frameworks, including the 1948 Universal Declaration of Human Rights and the 1950 European Convention on Human Right, fall short and are unable to sufficiently protect users of neurotechnologies against these mental privacy risks, and recommendations on the development of so-called ‘neurorights’ have been made to address these shortcomings^9,39–41^. Neurorights may be defined as “the ethical, legal, social, or natural principles of freedom or entitlement related to a person’s cerebral and mental domain; that is, the fundamental normative rules for the protection and preservation of the human brain and mind”^42^. In addition to these suggestions on the development of a legislative framework for the protection of mental privacy, others have also proposed practical design responses to address these concerns, including built-in mechanisms to promote user-control. For speech-BCIs, for instance, ‘veto control’ mechanisms that allow users to block the externalization of speech are a topic of discussion^43–46^. Still, it needs to be explored what control mechanisms are suitable for specific neurotechnologies (e.g. a continuous speech-BCI), and how benefits of additional control mechanisms are balanced against the efficiency of the device.

### Strengths and limitations

Incorporating stakeholder perspectives can aid understanding of how technologies are used and what their effects in practice may be and is needed to anticipate ethically relevant aspects of these technologies^1,47^. Conducting these ethical analyses in early stages of technological development, when important morally relevant decisions are made that influence how a device is designed, what it does, how it is used and how it can be accessed, allow for the ethical development of these technologies^47^. To our knowledge, the present study is the first qualitative empirical ethical study including developers of three types of AI-driven neural implants, offering unique insights in the relations between challenges in various stages of the development of these devices and their ethical implications for future users and society. These insights contribute to a deeper understanding of these relations, which would be unattainable through quantitative methods such as standardized surveys and scales. The data presented here can help inform future ethical and policy decisions on the development of ethically robust technologies.

This study also has limitations. Though data saturation was reached, further research should explore these topics in more depth. Additional empirical studies including developers of AI-driven neural implants may identify additional relevant ethical implications. Furthermore, relevant differences may exist between the ethical implications of brain-decoding and brain-stimulating AI-driven neural implants. These differences have not explicitly been explored here.

## Conclusion

The convergence of AI technology with the VNI, CI, and speech-BCI offers important opportunities to improve the quality of life of individuals living with blindness, deafness, or LIS. At the same time, this convergence also introduces new potential ethical implications that should be addressed.

Our study has shown that a tension arises between potential benefits of AI in these devices in terms of efficiency and improved options for interpretation of complex data input, and the potential negative effects on user safety, authenticity, and mental privacy. While a well-functioning device would increase independence and therefore promote users’ autonomy, the potential negative effects may simultaneously harm users’ autonomy. Though important suggestions have been made to mitigate these issues, including recommendations for the development of neurorights and mechanisms for improved user-control, more ethical analysis is required to further explore this tension.

Moreover, our study and other empirical studies involving developers of BCIs^10,11,18^, have shown that there is a need, as well as support for the inclusion of potential end-users in the development process of these neurotechnologies. Additionally it has been argued that to address the AI ethics concerns, voices of those using AI and those impacted by AI’s decisions should be included^3^. Empirical studies including potential users of AI-driven neural implants would be a valuable next step in mapping potential ethical implications of these novel technologies and could contribute to aligning the direction of development with user benefit.

### Supplementary Information

Supplementary Tables.

## Data Availability

The data supporting the findings of this study are not publicly available due to their containing information that could compromise the privacy of research participants. De-identified data are available from the corresponding author upon reasonable request.
