# Supplementary material for: Developer perspectives on the ethics of AI-driven neural implants: a qualitative study
Source: Sci Rep. 2024 Apr 3;14:7880. doi: 10.1038/s41598-024-58535-4 (PMC10991497; doi:10.1038/s41598-024-58535-4)
Supplement: Supplementary file 1 — Supplementary Tables. [file 41598_2024_58535_MOESM1_ESM.pdf]

Developer perspectives on the ethics of AI-driven neural implants: a qualitative study

Odile C. van Stuijvenberg[1], Marike L. D. Broekman[2,3], Samantha E. C. Wolff[5,] Annelien L. Bredenoord[4] & Karin R. Jongsma[1]

Author affiliations:
[1]Department of Bioethics and Health Humanities, Julius Center, University Medical Center Utrecht, Utrecht University, 3508 GA, Utrecht, The Netherlands
[2]Department of Neurosurgery, Haaglanden Medical Center, 2512 VA, The Hague, The Netherlands
[3]Department of Neurosurgery, Leiden University Medical Center, 2333 ZA, Leiden, The Netherlands
[4]Erasmus School of Philosophy, Erasmus University Rotterdam, 3062 PA, Rotterdam, The Netherlands
[5]Netherlands Institute for Neuroscience, 1105BA Amsterdam, The Netherlands

Correspondence to: Odile van Stuijvenberg, MA, MSc
Full address: University Medical Center Utrecht, PO Box 85500, 3508CA Utrecht
E-mail: o.c.vanstuijvenberg-2@umcutrecht.nl

Supplementary Table 1: Topic list and questions

Topics	Questions
Expectations and hopes regarding the [device]*	1. When would a [device] be "successful"/a success for you? Why?<br>- How does this compare to what you would consider success in the broader field of neurotechnology?<br>2. What functions would a successful [device] have?<br>3. What do you think the impact of the [device] could be on patients' lives?<br>- If we compare this to other neurotechnologies, is this effect specific for the [device]?<br>- What could be the effect on society, including non-patients when the [device] is more broadly used?
Design choices to be made	4. In your opinion, are there any important choices that are still open and need to be made in the design of the [device]? For example:<br>- How do you consider the (possible) switching between functions/functionalities/settings?<br>- What do you think about the more aesthetic/practical design of the [device]?<br>- What is your perspective on the possible use of AI with the [device]? How does this perspective relate to your views on the use of AI in the broader field of neurotechnology?<br>- Are there any other decisions that need to be made?
Concerns and risks	5. What are the (major) concerns or risks you foresee for the [device]?<br>- Technical challenges?<br>- In the clinical translation (to the patient)?<br>6. These concerns, are they general concerns (for neurotechnology) or are they more specific for the [device]?<br>- In the broader field of neurotechnology, where do the greatest concerns or threats lie?
Conditions for clinical translation	7. When do you think would be the right moment to move to first-in-human trials? / When do you think it is acceptable to initiate clinical translation to the first use in humans? Are there any particular conditions that must be met?<br>- For instance: What are the ideal candidates for a trial?<br>8. When would you consider a (FIH) trial to be successful?<br>- When is it is not a success?<br>- What could be the main risks involved?<br>9. When would it then be the right moment to allow the [device] to be more broadly implemented in clinical practice?
Potential impact on field of work	10. What impact do you think the [device] could have on science and your work?<br>11. How do you view the involvement/influence of Silicon Valley?
Remaining questions/remarks	12. Is there perhaps anything else you would like to say, that you feel is important to mention or that we may have forgotten to ask?

*The device of topic in each of the focus group (i.e. the AI-driven visual neural implant, the AI-driven cochlear implant and the AI-driven speech-BCI), can be entered in this topic list under "[device]".

Supplementary Table 2: Illustrative quotes on design aspects

Number	Argument	Illustrative quote
1a	The speech BCI will have to beat the current gold standard eye-tracker technology in terms of speed.	"Well, we have to beat the eye tracker, which is... that's already quite a high aim, I think, currently. Because eye trackers, of course they're way slower than the way in which we communicate, but still, from a BCI perspective, I think eye gaze and eye tracking based communication is much faster than what is currently possible with existing BCI." [Exp.4_1]
1b	The AI-speech-BCI could provide improvement over current technologies by targeting a population that are unable to use current technologies.	"I think that for those who are unable to use conventional assisted technology in an adequate way and in all situations, I think that's the initial target population that we're currently aiming for." [Exp.4_1]
1c	The AI-CI could provide an improvement over current technologies when it works just as good as the current system, but is more future-proof.	"So that you then say, okay, instead of the old classical methods, we will move to something that is self-learning or externally steerable, so that patients can upload their own algorithm and that in this way you actually generate a platform for the future into which we can put new things. And then you might even say, well, it's just as good as the current state of the art, but we can do a lot more in the future to further steer development." [Exp.3_2]
1d	The ultimate goal is to have an AI-CI that knows what the user wants without the user's intervention.	"if we want to get to the ultimate goal [to] be successful [...] you actually want it all to work without user intervention. Which then is more indeed about how do you figure out what someone wants to hear and in what context as well." [Exp.3_5]
1e	For the speech-BCI there is room for improvement in the user-interface	"The user interface, basically, there's still a lot of design... room for design, yeah." [Exp.4_1]
1f	It is important that a speech-BCI functions adequately in all situations and all times of day.	"And in addition to that, I think that that functionality, adequate functionality in all situations and at all times of the day would be, I think, important." [Exp.4_1]
1g	The speech-BCI can also be improved in terms of versatility and accuracy in order to allow for more natural communication than the eye-tracker method.	"Next to speed I also, I guess, wanted to mention also the accuracy, as well as the versatility of the system. Because, I guess, if we talk about eye tracker, then is that mostly yes and no kind of responses? At least I know that there's some technology, current technology that's based on the yes and no type of communication. And of course, they want to bring it a step further and help people communicate in a more natural way. They could actually communicate with sentences, words and something that comes more from them rather than just yes and no responses, which is, I think, a very big step forward too." [Exp.4_5]
1h	Involving feedback of users in the design process of the speech-BCI contributed to the design and was good for the ivory tower way of thinking.	"The interesting experience we had is that, as far as the human interaction, was that the feedback of the users... so really, they were designing with us. We changed a lot of things, we added features in the software which they requested, and that helped a lot. […] That was very good for our ivory tower way of thinking." [Exp.4_3]
1i	There are many risks involved in brain decoding and in brain modulation, which has to be done safely and as intended by the developers.	"I think in our case there's tons of risks because we have to read information from the brain, and it has to be done accurately. And then we have to modulate brain activity, and it has to be done accurately. And somehow this has to be done in a way which that's been designed safely and tends to behave as we would like it to do." [Exp.4_2]
1j	Fundamental research on the underlying mechanisms of brain-reading and brain activity modulation should be done to mitigate risks on unforeseen issues.	"And there can be foreseen circumstances, activities in the brain that we didn't foresee, and that can cause issues, so there has to be... That's something that we are not doing, because we are really preclinical, but that's something to take into account, all the protection measures that have to be in place. […] Well, there should be more, I think, fundamental research on the mechanisms." [Exp.4_2]
1k	The lifetime of the electrodes is uncertain.	"Well, of course we have talked before about the lifetime of those electrodes […] I think that is also still a matter of concern." [Exp.2_4]
1l	More information is needed to make several design choices.	"Just the same, we need more information about thresholds, about how many electrodes should we stimulate to provide a useful sense of vision, about the technology. For instance, right now one of our main I will say limitations is we have no telemetry. So we have to develop that wireless system. For the future it's a need, exactly a need." [Exp.1_1]
1m	Open questions remain on how psychophysics and stimulation relate to perception.	"I think there are a lot of things to do also in the psychophysics, how stimulation relates to perception. Especially when you're stimulating with multiple electrodes at the same time. I think that's not completely clear, although there is a lot of research in humans and animals in the past. But also yeah, since the project was the first, like a high-resolution implant, let's say, a lot of questions were solved, but we have a lot of questions more." [Exp.1_3]
1n	The biggest challenge for making AI-driven systems better is the breadth of input.	"But […] the biggest hurdle is I think, a schoolyard with screaming children is just really different from a meeting with your colleagues. Just the spectrum and the type of speech and what you want to hear, so I think that, that

		breadth of input, that's where the biggest challenge is for AI-driven systems to make it better." [Exp.3_2]
1o	Switching between scenes in a complex auditory situation may be a challenge for the AI-CI.	"I think indeed that becomes a very big problem, well, now you have all these scene selectors in there, but switching the scenes you have and determining what you want to hear. At one moment you want to talk to someone during a concert and at another moment you actually want to hear that music. […] And so you have these very complex situations with switches of what you want to listen to, how you build that into such a system. […] But just the complexity of speech and noise, music, which all need their own setting and optimisations, how you get that right." [Exp.3_2]
1p	Algorithms may function perfectly in one language but not in the next.	"We may end up with the perfect AI for the Netherlands and then it doesn't work in Germany" [Exp.3_2]
1q	Complete noise-reduction in a noisy environment may also lead to an undesirable distortion of voices.	"We had the example with Clear Voice. Clear Voice is a way to, quite statically, remove background noise and there were patients and so they didn't hear that they were in a noisy environment anymore and they started to complain that the voices were distorted. And, but the moment you put the noise on, they had, oh, but this is speech and noise, yes, then I understand it's a bit distorted. Then they accepted it, but because in some situations they didn't realise there was noise anymore, they started complaining about the distortion and they didn't think that distortion was accurate, they didn't approve of that. And, so that's kind of if you go too far that patients start hearing distortion without knowing why it's distorted. And so if the systems work too well then you're actually, well, yeah, you're shooting yourself in the foot." [Exp.3_2]
1r	An individualized algorithm may be desirable yet currently unfeasible.	"... so a trained algorithm per location almost, you could say I'm going to make an algorithm per individual, but that' s, yeah, but that's distant future." [Exp.3_1]

Supplementary Table 3: Illustrative quotes on challenges in clinical trials

Number	Argument	Illustrative quote
2a	Safety is the main point of clinical trials.	"For sure safety is concern number one for everyone. And that is actually one of the... always the main points in the trials, the clinical trials. The main matter to measure and to prove, there rest are secondary." [Exp.1_3]
2b	The biggest safety concerns relate to type of physical harm caused by brain surgery.	"Well, to elaborate on the safety, the biggest concern, of course, would be disability or death. If there is excessive bleeding in the brain or if there's damage or degeneration of different brain areas... Yeah, or risk of infection." [Exp.1_4]
2c	A high resolution in the AI-VNI would imply more electrodes and thus more invasiveness.	"And also, it depends on the size of the prosthesis. The thing about the visual system is it's a system that depends on quite high resolution to transmit meaningful information, which would imply more electrodes, which would imply more invasiveness." [Exp.1_4]
2d	The rehabilitation period of a visual implant can be demanding, and the life phase of a participant may influence what other commitments they have and how willing they are to give them up.	"When you hear how burdensome or how demanding it can be, then it's good to know: God, does someone also have a job and to what extent do you want it to have an impact on the everyday things that someone does and to what extent is someone prepared to either give those up or fill them in differently? So of course, that what life demands varies enormously from one stage of life to another." [Exp.2_3]
2e	Speech-BCI trials require high levels of motivation in from participants as they requiring a large time-investment.	"So, in our case, because we need so much data, especially if we go to AI-like models, then you need a lot of data to get accurate models. So, they will have to, it's a large time investment for them, it's two mornings or afternoons a week at least, and that's for at least a year and possibly many years if it goes well and we get better and better results. They have to have the motivation." [Exp.4_3]
2f	Patients with progressive ALS are highly motivated trial participants.	"I think the motivation is strongest in the disorders that are very progressive, like ALS. We also have the slow progressive disorders. I think ALS patients are a very specific group, because usually they are in the midst of their life, it's a sudden event, and they have a very short horizon. So, in the few years to come, they are willing to contribute to science or to look for solutions for themselves. But I think in this group motivation is very high. We also see it in medicine trials. They're always motivated for everything." [Exp.4_4]
2g	Willingness of participants to undergo invasive ventilation and opt for reanimation reduces risks around surgery and shows motivation to continue their lives, which is desirable for lengthy speech-BCI trial.	"our clinical trials are very long, we aim for at least a year, but we are very happy if they continue in the study for many years. […] We, as scientists, want to feel both for the risk... It's about invasive ventilation, so if patients are not willing to go in to have invasive ventilation, then the risk of surgery may be too high. If they're willing to do that, then we could take the chance. […]. I think that shows the will to, the motivation to continue for a long time. And that's important. […] They also have... I'm not really sure, so patients who want to get reanimation if something is happening, that also shows that they are very motivated to continue their life with all the limitations they have." [Exp.4_3]
2h	Media coverage of neural implant trials should be streamlined and participants should be aware of the consequences.	No, what it is, apart from trying to streamline it, the patient should also be very well know what the possible consequences could be of if you start this, the media coverage and in what form and so on." [Exp.2_1]:
2i	It is important that trial participants understand that the AI-VNI is still research, and not clinical treatment. It is a problem that most people expect to see again.	**"**OK, we are looking for, first of all, people have to fully understand that this is still research. It's not a clinical treatment. […] our main problem is to find people that fully understand that this is still research. Because most of the people expect to see again. That's a big problem." [Exp.1_1]
2j	For the AI-VNI an extensive screening process including psychological tests regarding expectations would be important.	"Yes exactly. An extensive preliminary process with psychological tests seems to me to be a very important one. What may you expect from someone, what can you expect from someone, how do they feel about it?" [EXP.2.3]
2k	The use of language in communication with patients is important for the management of expectations.	"Well, beyond what a patient can and may expect from the chip, use of language is incredibly important. Because the bit about 'seeing' everyone interprets differently. We also used to say: it's not seeing, but it's visual perception, so perceiving. We constantly correct that. But in our eyes, it wasn't seeing, and I don't know how it is for someone else." [Exp.2_1]
2l	It is important that a potential participant reasonable and accepting in their current situation, as they might return to this situation after the trial ends.	"I think it's important that a patient, the current situation that they're in, that they're solid in that, because there is indeed a return to that situation again eh, when we talk about the temporality of it. And the moment that the desire otherwise becomes so great that it is all going to change so much and that it is wobbly and not anchored, yes, […] there has to be a solid foundation in someone, in a reasonableness and acceptance. I hate that word so much by the way, but at least the situation visually as it is for this patient, that that is calm." [Exp.2_3]
2m	The planned explantation of the AI-VNI has to be communicated clearly to trial participants.	"Well, the very first time [name principal investigator] said: 'yes, but at some point we will have to take out that chip again, maybe after three months.' Well, at least it landed with us that we were like: 'Hey, take it out? Okay...' So it hadn't sunk in with us. […]. Well, of course, that has to be

		communicated very well to a patient that it's for a short while, and then we can learn from that and go on developing." [EXP.2_2.]
2n	It is important to make clear to the patients what (long-term) consequences the implantation of a device can have and whether it can be removed.	"At one point in the end, it was also said with the retina chip that the chip could not be removed from the eye, because it is attached with some kind of nails and that that is actually very difficult to remove. That was not well said, made clear, or received by patients, with the result that one person indeed found it very annoying, or finds it, because they still have pain in the eye. Because they thought: it can come back out. So that turned out not to be the story, and with very big consequences. […] So that also that is made very clear to the patient what consequences it can have and how often you can put it in and take it out." [Exp.2_1]
2o	Benefit for participants of trials for the speech-BCI cannot not be guaranteed.	"Well, I can't give 100% guarantee. […] We will never do that." [Exp.4_3]
2p	It is preferable to give speech-BCI trial participant benefit from study participation, making patients with progressive diseases good candidates.	"We actually prefer to give patients the benefit. So in that sense we prefer progressive disease, because we feel we can do something for these particular individuals." [Exp.4_3]
2q	Patients have to realise that they are participating in a pioneering phase of research and that there is always the risk that a trial is ended because of risks or a lack of effectiveness, and that this is quite common.	"It's important that patients are very aware what they are starting when they get into a trial like this. And it's the same for medicinal products. Medical trials stop because the effectiveness is not good enough or the side effects are too big. There's always a risk, and people have to realise that yes, they will be some kind of a guinea pig. Well, that's always in the pioneer phase. We have to realise, and we have to be clear to patients, that we are in a pioneer phase, that this might happen. In our organisation it's quite common that these things happen, patients know." [Exp.4_4]
2r	Even if trial participants for a speech-BCI may not benefit from the device, they can still get a good feeling from participation as they are doing something helpful for society.	"But also, we learned a little bit from our studies, but also from other studies where it was clear there's no benefit, but it is still a large time investment, that they see it as a kind of job or as a kind of doing something for society, which gives them a good feeling that they are doing something helpful for society. In that sense the motivation is on different levels, and that is very interesting" [EXP.4_3]
2s	There are stories about tetraplegic patients for whom it was a great shock that after participating in a trial for an exoskeleton that they had trained for to control, they had to stop using it	"Can I also mention something about the technology abandonment? At least some of the stories that I heard come also from the other side when, in tetraplegic patients and tetraplegic users, they for example invited people to participate in a clinical trial and it took people, participants, a lot of effort to – something that [Exp.4_1] also mentioned – to actually train to control that exoskeleton. But then, after some period of time, the clinical trial was over and they had to stop using that technology and that was a huge change for the person, a huge shock, because they had to give up something that they've worked so hard on for such a long time, and then suddenly it was over. And it was a huge thing." [Exp.4_5]
2t	Post-trial abandonment of technology goes against the declaration of Helsinki, which is why provision for post-trial access are built into the protocols.	"It goes against also of the Declaration of Helsinki. So, in our protocols we have post-trial access, provisions for post-trial access built in." [Exp.4_3]
2u	The requirement for post-trial access to technologies is a grey area as it depends on what trial participants agreed to on the consent forms.	"in the media it is portrayed as though the company somehow screwed them over, […] that it's a bit of a grey area, because we don't know exactly what they agreed to in the consent forms. And I expect that for all these first-in-human studies, they knew and they were told many times, and asked many times whether they understood that this may not benefit them at all." [Exp.1_4]
2v	Arrangements for continued access to hardware should be made in case of bankruptcy, similar to those currently present for cars.	"Naturally, Second Sight had the obligation, which of course pulled the plug on everything. Yes, you see what happened to [name retinal chip user]. Well, luckily some were still able to scrounge things together here and there. But you see, on the internet there has been a whole riot about it from all over the world. And rightly so I think, and rightly so yes. That's something you should be able to cover somehow. With cars, I understood that if the supplier goes bankrupt, parts have to be supplied for another 12 years. And it doesn't really matter by whom, but I think there is some kind of agreement about that." [Exp.2_2]
2w	There are challenges in providing long-term post-trial access support.	"Yeah, because the technology, of course, changes, so you can then, as a development team, move on to more upgraded versions, better performing versions of that technology, but you still have to provide support for earlier versions, and indeed, that can become very difficult." [Exp.4_5]
2x	It may be an option that insurance to cover explantation of neural implants is taken out at the time of implantation.	"But would that be solved then if you have the implant, if you decide to have the implant, you also always buy something or insurance pays for the explantation or the removal of that implant. So yeah, whatever, if something happens, the whole implant will be, there will be funds to remove the implant. Does that solve it?" [Exp.1_2]
2y	Researchers are trying to develop a support network for Second Sight	"there are now researchers who have worked with Second Sight patients, and they are trying to kind of have a community, pull a community together

	patients who still use the retinal implant, yet legal issues prevent them from being able to fix these medical devices.	as a support network for these Second Sight patients. But the thing is that both Second Sight and the researchers are not actually even allowed to... As far as I understand, they're not even allowed to upgrade or fix the implants because this is a medical device. So even if they know how to fix them, you know, like the electronics, they are legally just not allowed to do anything to the implants. So everyone's hands are tied." [Exp.1_4]
2z	Not all patients can have the implant removed, because of age or medical conditions.	"I could definitely imagine situations, or now the patients are too old, or they developed some medical condition, and then they can't have it removed or something and then they are stuck with it." [Exp.1_4]

Supplementary Table 4: Illustrative quotes on users and society

Number	Argument	Illustrative quote
3a	Regaining independence in users is the most important aim of the AI-VNI.	"Independence is everything in my opinion" [Exp 2.4]
3b	It would be a success if the AI-VNI can contribute to solving some of its users' independence-questions.	"Well, [Exp.2_4] actually hits the nail on the head. I think when people, they have their questions and their independence questions, and I think if the chip, the prosthesis, can provide for some of that, then you already have a reasonable success." [Exp.2_2]
3c	A blind advisory expert by experience, listed experienced difficulties, including finding the way, knowing whether the person they were speaking to was still there, and recognizing if a seat is empty.	"So we have contact with for example an advisory expert by experience who made a list of the different areas in which as a blind person he experiences difficulties. And he categorized them... from things very obvious, like not being able to find your way to the bathroom, to things like not knowing if the person you're talking to is still there. So, he had this experience where he was talking to someone and then, after some time, he realized that they had gone. So that's very annoying. Or even just knowing whether a chair is occupied if there is someone already sitting on it or not, or which way the chair is facing." [Exp.1_4]
3d	Being able to choose when to get in contact with others would be beneficial for AI-VNI users.	"Well, not being dependent on others […] so that you can choose for yourself when to get in contact, without it being decided for you." [Exp.2_4]
3e	The AI-VNI could allow users to be more socially engaged with others because they can then also observe things and discuss them, like sighted individuals.	"I don't quite know what to call it, but a piece of engagement. So precisely because you can observe things, that this allows you to join the conversation about that which your colleague, your family member is also talking about, because then you have been able to observe it yourself. […] So that with that, that you are more of an equal than very black and white said the blind and the sighted. But that with that, therefore, you get closer to each other." [Exp.2_1]
3f	An AI-VNI could improve feelings of isolation in blind persons.	"Yeah, I think probably the matter of the social part is very important. The isolation that you can feel if you're blind, right?" [Exp.1_3]
3g	Small improvements in visual function can positively impact a blind person's self-esteem.	"Agreed. I still think that at times, for us these seem to be very small changes, that at the moment you can do something visually yourself or that something contributes to less dependence because you are visually stimulated after all […] for us that may not seem so substantial, but I have experienced that sometimes it can be in very small things that a person really, well, does something with the self-esteem." [Exp.2_3]
3h	An AI-VNI could improve a sense of agency in its users.	"And I guess it's everything, almost everything related to agency, right? That you feel you are an able person." [Exp.1_3]
3i	An AI-CI could lead users to more proactively take part in life.	"and all these hypotheses always said if you have a better technology the people that use it actually go more out, are more and more proactively taking part in life." [Exp.3_3]
3j	As currently only very good CI-users can participate in meetings, regaining function will give many CI users a better perspective in the job market.	"It varies per patient, but for most people, look, also, impact on life is, for example, can you function in a work situation? And there you notice that there is still a lot to be gained and [that] the very good CI users can participate in meetings and the like, but as you have also noticed at the, well, the users' association, the slightly lesser users will still use type interpreters, they […] are still missing things no matter how hard they try. […] that has quite an impact [...] if you can […] function again in that kind of situation, it just gives you a total, yes, a better perspective on the job market." [Exp.3_1]
3k	Regaining the option to communicate for people who have lost the ability to speak, may have a strong impact on their quality of life.	"we know that […] adequate communication is really important for the quality of life of people. And at least for those who are tetraplegic and have lost the ability to speak. So regaining that option, I think that could have a very strong impact on the quality of life there, also their ability to be autonomous and have... you now, be able to control their life, basically, independence, etc." [Exp.4_1]
3l	The speech-BCI is really a success when participants can continue to use it at home.	"Our feeling is that it really is a success if they continue using it at home. […] Even after the trial, yeah." [Exp.4_3]
3l	The initial target population of the speech-BCI is individuals who are unable to use conventional assistive technology, like many late-stage ALS patients, for whom prospects on continued abilities for communication may improve quality of life and considerations on the desire to remain alive in case of disease progression.	"I think that for those who are unable to use conventional assisted technology in an adequate way and in all situations, I think that's the initial target population that we're currently aiming for. I think there was a study that was published in 2020 by a Japanese research group on a very large cohort of people with ALS, late-stage ALS, and about only 15% of those reported that they had no trouble using the current conventional assisted communication technology. […] And in the end, I can imagine that... But that's really speculative. At some point, if people with ALS for example, progressive neuromuscular disease, they know that communication will not be an issue; if we do our job really well, you know, the considerations related to quality of life and the desire to remain alive when tracheostomy, invasive ventilation comes into play for example, those discussions may at some point change, perhaps. But that remains to be seen, if that's indeed the case." [Exp.4_1]

3m	The aim is for the AI-CI to figure out what someone wants to hear and in what context, without user intervention.	"[…] what you actually want [is for] it all to work without user intervention. Which then is more indeed about how do you figure out what someone wants to hear and in what context as well." [Exp.3_5]
3n	Mistakes in predictive scene selection for the AI-CI would hinder users rather than help them.	"Yes. Look, what you now have with scene selection, for instance, if the wrong scene is selected by your processor, yes, you are actually helped backwards instead of forwards. And, yes, those scene selection algorithms are very generic and so in a traffic situation it can be very unpleasant if all the traffic noise is suppressed because just that car arrived." [Exp.3_1]
3o	In the AI-CI there is a tension between the advantage of automatic AI-based scene selection and the level of user-control.	"One moment you want to talk to someone during a concert and the next moment you want to hear that very music. And at the same time nobody wants to notice that the system is switching, but actually what normal hearing people can do, they can indeed put their focus on from one to the other. And so that you have these very complex situations with switches of what you want to listen to, how you build that into such a system. […] And it may well be that then you do have to give a user a choice, I think that works better then, that they have a sense of control." [Exp.3_2]
3p	Predictive models such as speech and language models used in the speech BCI may lead to autonomy issues when biases leak into the performance.	"speech and using language models in itself that people, or the computer, is going to pronounce sentences that are largely based on language models, but that were not specifically what the user wanted to express. So, there could be also a problematic autonomy issue there, potentially."[Exp.4_1]<br><br>"Yeah, that's what they call bias in AI, so any kind of biases that inherent to those models can also leek into performance, yeah." [Exp.4_5]
3q	With the implementation of AI in the VNI, it may be difficult for the user to know what the device may have missed.	"I think that raises a very interesting point, because if you use AI for the image detection, you kind of have some kind of important intelligence, right? But it's hard to learn for the user what intelligence your algorithm lacks. Whereas, if you just have a Canny edge detector, you know, kind of, and I think you can learn as a user, what the benefits are and what is typically missed by a Canny edge detector. But that's a different discussion." [Exp.1_2]
3r	CI users who currently function well will react negatively to losing control to an AI in a new AI-CI, when users who currently perform worse in noisy environments will likely benefit more from it.	"...especially people who are in control of that environment with their current technology and don't get stuck communicating at important, for them important moments, yes, they don't have such a need for that and they have, and they will react negatively to it. […] whereas I think most of the users who […] as soon as noise comes in start performing worse, that you can draw those in. So I don't expect so much that you're going to make top performers better, but I think you're going to bring up the middle and the bottom." [Exp.3_1]
3s	Preferences regarding the amount of user-control may differ between individual users and it is therefore important to offer the technology which accommodates these varying preferences.	"For example, I have one of those people, and he studies AI then and he, and, yes, he likes to give feedback or play with it himself. We also have an audiologist here, who is quite old, but he is adjusting himself and he wants to have total control. But, for example, older people who are like, please let it just be completely automatic, I don't get this, I don't want this, I want to push a button on or off. […] with this entirely new technology […] I think it is ultimately very important how you are going to offer it. If you're going to offer it as one package on or off, say, the system does everything itself that's really not going to please some people, because they want to keep that control." [Exp.3_6]
3t	Reliability and accuracy in the interaction with the brain is important for safety and trustworthiness of the AI-VNI.	"in the trials […] the second thing, I will say, is reliability. Because it is very difficult to interact with the brain. What will happen, for example, if you present, if you create perceptions in a person that are misleading or are not complete? And that creates either a safety issue when navigating, or you know, you can fall or bump on something that your algorithm didn't capture, or your stimulation algorithm didn't make possible for the person to see. The other thing, I will say, is trustworthiness, because if I'm a blind person and I realize sometimes the reliability of what I'm seeing is not very good, I would be concerned about that. And this related to the human-computer interaction in general. So, if you are using Google Translator and your translator is 95% good, still it is a very, very bad translator. It needs to be 99.99%, if not, you get kind of mad at it: This thing doesn't really work. So, the standards that we have nowadays are very high. So, what about if this related to what I'm seeing? So, reliability in the functioning, in the perception, I think that would be a big issue." [Exp.1_3]
3u	Failing algorithms in neural implants could introduce questions on responsibility.	"Yes. Failure of algorithms I think is a bit trickier. Huh, you see that in self-driving cars already a lot, of course, for example. Who takes responsibility for the fact that the algorithm makes a wrong choice there? And yes, now I don't expect that to become a very big problem with cured blind people. But yes, of course there is a risk in that. It's not for nothing that those manufacturers are all so afraid of it." [Exp.2_4]
3v	If you let the AI for the AI-CI train on a large external server to develop an individualized AI, this would allow for	"But depending on how you, say, implement the AI, if you indeed, say, switch it on, hook it up to a very large server somewhere [...] And you let those things train, train, train, train, train, train, for each CI user separately,

	personalized care, but would also mean storing and processing all surrounding sounds of a user, including conversations with others, which could imply GDPR issues.	[…] that would work very well, because then you get that personalized care. But that does mean that the surrounding sounds are all stored, also the conversations, also the doctor's visits, everything is put on that Google server or I don't know what happens. So yes, then you do get ethical, GDPR things you have to think about, but that again depends on how you ultimately implement that." [Exp.3_6]
3w	Even if it is not necessary to record full conversations for scene selection in the AI-CI, a sound fragment needs to be recorded which may cause privacy issues.	"No, no. No, no. There's certainly a, and it doesn't have to be that all conversations are recorded, but if you have to characterize the situation through a remote server is that situation, you need a fragment of that. And there, of course, you do have issues with privacy." [Exp.3_1]
3x	As the speech-BCI contains a software, there is a risk of hacking, and when outsourcing data in language models for communication, or when interacting with other resources, there are additional data privacy risks.	"Yeah, I think so. So yeah, that would be one of the risks of potential hacking, because it is software. […] Another risk I could see is moving forward if we start talking about edge devices and how everything potentially could be computed or processed on a cloud system. That is, of course, additional safety risk. Use of external resources, like we're talking about language models for communication or something else that is also outsourcing data or using some kind of interactions with other resources and potential risks. So yeah, everything that deals with information safety has to be mentioned as a potential risk, privacy risk." [Exp.4_5]
3y	A societal risk could be if the technology is used in another way than helping patients, e.g., if a speech BCI people is used to listen in on the user's thoughts or inner monologue.	"Let me discuss the societal risks […] That is something we should discuss now, we do, but that is really more in the thinking of what, how could this technology be used in a way that is not meant, as we do, to help patients. If we do a speech BCI, we'll be able to listen in in other people's thoughts or internal monologues or something." [Exp.4_3]
3z	Using an AI-CI auditory data of conversational partners is used.	"Yes, I agree. But there is another aspect to it, if I as a CI user decide that my conversations may be used, my conversational partners know that and may they, may their data be used, because I need it to filter." [Exp.3_1]
3aa	Data recording and online processing of (auditory) data already happens for daily-used consumer products, such as smartphones and other mobile devices.	"But, yes, let's be very honest we all know that they can remotely turn on the microphone of your mobile phone and listen in. So, yes, and we all have those on the table as well, so in practice, it is indeed, there is something to this, you make the choice and if you want to use it then some of your conversations will have to go through the world. But I think it's also a bit of a utopia to think that nothing happens now." [Exp.3_2]
3bb	Facebook gets sued about privacy issues surrounding data processing. It is interesting to see how to do this ethically.	"if this is about ethics, you […] have Facebook that is just being sued for precisely this kind of thing, so I think it might be interesting for you to think about precisely this kind of thing in terms of how to do it properly. Rather than getting a Zuckerberg stamp on the forehead, because well, yes, he doesn't have a very good name anymore." [Exp.3_6]
3cc	Laws are tighter for medical devices like the AI-CI than for consumer products.	"Because we are still a hearing, I mean, medical device and that's why the law is basically tighter." [Exp.3_3]
3dd	Streaming audio data of people who are identifiable is not allowed without their consent, therefore only parameters of auditory data are transmitted in the codex of the CI, rather than audio signals.	"But that's why you have the laws in place, right, that also now when you are on trials you are not allowed to collect any data which might identify the person around you. So it's never that you transmit audio signals, it's always that parameters get transmitted in this codex, so that's what is already kind of taken care of by law. So…[…] that's something which can be done, but by law it's not allowed to basically use audio streaming data where you cannot make sure that people who did not get, put their consent in place are identifiable." [Exp.3_3]
3ee	Apart from legal interpretation, the perception of privacy is important.	"it is perhaps not only about what is possible in terms of legislation but also to some extent about the user's perception. If they feel that their privacy is disappearing, that can of course give a negative aftertaste to this" [Exp.3_5]
3ff	Acceptance of the technology as a good treatment option is important, not only by users but also in a broader sense by clinicians that prescribe it and society at large.	"For me, if BCIs or neurotechnology would be embraced, success will be defined for me when it's embraced by users, but also by clinicians and society at large. Because I think that showing that... if with all the precautions and ifs and buts that are associated with that, users say "OK, this is useable for me", then still, I think, there's a hurdle to take […] before we can define something as a success. And that is that also the rest of society and clinicians who are going to be advising on these matters are going to actually prescribe it. And that it is kind of accepted in a broader sense, I think for me that defines success." [Exp.4_1]
3gg	Societal acceptance is important for generating funding from companies and investors.	"I think maybe societal acceptance is another thing. I think one of the key challenges is having companies and investors who are willing to step in and provide the resources, because they and the patients and society think that this is a good enough investment. So I think this is one of the reasons that previous companies have failed, the technology is still not mature enough. So that's a kind of financial/business benchmark." [Exp.1_4]
3hh	Developing a VNI would identify a solution for people who are disadvantaged and could provide hope	"I think hope is the big one, that we really identify some solutions or therapies for people who are among the most disadvantaged in society. That gives sighted people hope that if they end up in that position, that they

	for those who may find themselves in this situation later in life.	at least have something to look forward to and that other people are also trying their best to alleviate difficult conditions." [Exp.1_4]
3ii	There is a risk of societal disappointment caused by big promises of companies working on BCIs, as the progress will be slow and limited in regards of target population. This could negatively impact societal and patients' acceptance.	"I see also another societal risk and that is that, you know, because of this study and other studies and big promises made by some companies that are working on BCIs, I think the hopes are high, they're growing. And progress will be slow, because much of the validation studies will be limited to, you know, one, two, three participants at most. And also the initial target population is very small, so progress is most likely going to be much slower than what society may expect based on all the news outlets, etc., etc. So, I think that there's also a risk of societal disappointment or so, at some point, which may affect in the end the acceptability or the acceptance of society and patients of this kind of devices." [Exp.4_1]
3jj	Availability of neural implants may be at risk as their small target group, small study sizes, high costs and effectiveness compared to other assistive technology, may make limit chances of reimbursements.	"There's also a risk of hope and availability. In the end, when the system is ready, it has to be reimbursed or not. And we have the problem of quite a small target group, small experiments, probably quite high cost, and the effectiveness of the system compared to the other assistive technology that's available. I think that's quite a risk. And it's also a risk." [Exp.4_4]
3kk	The aim to develop a successful AI-CI, that offers a significant improvement over current technologies, is primary one of cost-efficacy.	"This is more of a matter of costs than an ethical question I think. We think every little step you make is nice [...] but if you have to make a very large investment that allows ten per cent of CI users to improve 1% of the situations, I don't think [the manufacturer] is going to say 'we're going to design new processes for that". [Exp.3_1]
3ll	The AI-VNI could reduce societal costs by improving equal societal participation and access to the work environment.	"So, if people are able to participate more equally. […] I read some statistics that say that blindness causes a lot of costs in terms of healthcare, and also rehab support. So, if people are a bit more independent and maybe have more access to the work environment, that could also help society in terms of decreasing these costs." [Exp.1_4]
3mm	CIs can reduce societal costs by improving health, life-expectancy, work-years and social isolation and can improve mental and physical wellbeing, by allowing users to take part in society more.	"Reduce costs because people live longer, that's what it's all about, but the biggest impact of the CI itself is just simply reduce costs of the tech system. Because people are healthier, live longer, can work longer and have a better life, healthier life, which means less hospitalisation, less risk of falling, less risk of social isolation. So this mental or this physical and mental wellbeing, I think that's one of the biggest impacts of CIs. That you have people who can basically take part in society the more we help them to support them in the situations they need. Which is work, which is at school, I mean, if the things work better at school and universities then you have also new, next generation of people that can work and contribute to society. So I think that's, on the bigger scale that's, it's cost reduction of health costs and so on, that's most probably the biggest impact on the society." [Exp.3_3]
